# “Long COVID-19” and viral “fibromyalgia-ness”: Suggesting a mechanistic role for fascial myofibroblasts (Nineveh, the shadow is in the fascia)

**DOI:** 10.3389/fmed.2023.952278

**Published:** 2023-04-06

**Authors:** Shiloh Plaut

**Affiliations:** Department of Basic and Clinical Sciences, University of Nicosia Medical School, Nicosia, Cyprus

**Keywords:** SARS CoV-2, COVID-19, pain, myofibroblast, fascial armoring, fibromyalgia, pathogenesis, psychosomatic

## Abstract

The coronavirus pandemic has led to a wave of chronic disease cases; “Long COVID-19” is recognized as a new medical entity and resembles “fibromyalgia” which, likewise, lacks a clear mechanism. Observational studies indicate that up to 30%–40% of convalescent COVID-19 patients develop chronic widespread pain and fatigue and fulfill the 2016 diagnostic criteria for “fibromyalgia.” A recent study suggested a theoretical neuro-biomechanical model (coined “Fascial Armoring”) to help explain the pathogenesis and cellular pathway of fibromyalgia, pointing toward mechanical abnormalities in connective tissue and fascia, driven by contractile myo/fibroblasts and altered extracellular matrix remodeling with downstream corresponding neurophysiological aberrations. This may help explain several of fibromyalgia’s manifestations such as pain, distribution of pain, trigger points/tender spots, hyperalgesia, chronic fatigue, cardiovascular abnormalities, metabolic abnormalities, autonomic abnormalities, small fiber neuropathy, various psychosomatic symptoms, lack of obvious inflammation, and silent imaging investigations. Pro-inflammatory and pro-fibrotic pathways provide input into this mechanism *via* stimulation of proto/myofibroblasts. In this hypothesis and theory paper the theoretical model of Fascial Armoring is presented to help explain the pathogenesis and manifestations of “long COVID-19” as a disease of immuno-rheumo-psycho-neurology. The model is also used to make testable experimental predictions on investigations and predict risk and relieving factors.

## 1. Introduction

The coronavirus pandemic has impacted our world not only because of the acute phase and its mortality. “Long/Post COVID-19” is recognized as a new medical entity and resembles functional psychosomatic syndromes (1–4). The post-acute sequelae of COVID-19 is being diagnosed not only in those who developed severe acute COVID-19, but also in infected individuals who had mild and even asymptomatic cases (3). In current literature there is no consistent term for the post-acute sequalae of SARS-CoV-2 infection. In this paper, the term “long COVID-19” will be used.

Recent investigations indicate that most “long COVID-19” patients present with lasting symptoms that impact morbidity and quality of life, ranging from neuropsychiatric, cardiovascular, pulmonary, gastrointestinal, musculoskeletal, reproductive, immunological, otolaryngologic, and dermatologic symptoms, up to multiorgan systemic dysfunction (2). Studies have been reporting various incidence rates for “long COVID-19.” According to some estimates, long-lasting symptoms following COVID-19 affect as much as 76% of people at 6 months post-infection (1). The United Kingdom office for national statistics estimated that the five-week prevalence of any symptom was 22.1%, and the 12-week prevalence was 9.9% (1).

The puzzle of the similarity between “long COVID-19” and functional somatic syndromes is intriguing to many (1, 3, 4). A recent study (4) suggested a theoretical model (coined “Fascial Armoring”) with a cellular pathway to help explain part of the mechanism and pathogenesis of fibromyalgia-like and “functional psychosomatic syndromes.” Psychosomatic syndromes are suggested to be on a spectrum of a single medical entity (5), which may share a common rheuma-psycho-neurological mechanism (4). Fascial armoring describes the rheumatological facet of this medical entity. It suggests that these syndromes are driven, in part, by myofibroblast-generated-tensegrity-tension in the fasciomusculoskeletal system. This work offers a theoretical model with a cellular pathway to help explain the mechanism of “long COVID-19.” In light of the findings, the discussion proposes that fascia may have a role in the mechanism of “long COVID-19,” which may involve myofibroblast-mediated-biotensegrity-tension.

The paper is organized as follows: section 2 presents the hypothesis of this work. Section 3 presents the theoretical model of fascial armoring. Section 4 gives an overview of “long COVID-19” manifestations and SARS-CoV-2-related fibrosis, and a suggested mechanistic link between SARS-CoV-2 infection and fibromyalgia-like syndromes. Then, the discussion in section 5 provides an explanation of “long COVID-19” symptomatology in light of this theoretical model. Afterward, predictions of expected results of future investigations of “long COVID-19” and risk and relieving factors are presented based on the suggested model. Finally, section 6 is a summary and conclusion.

## 2. Hypothesis

**Figure 1:**
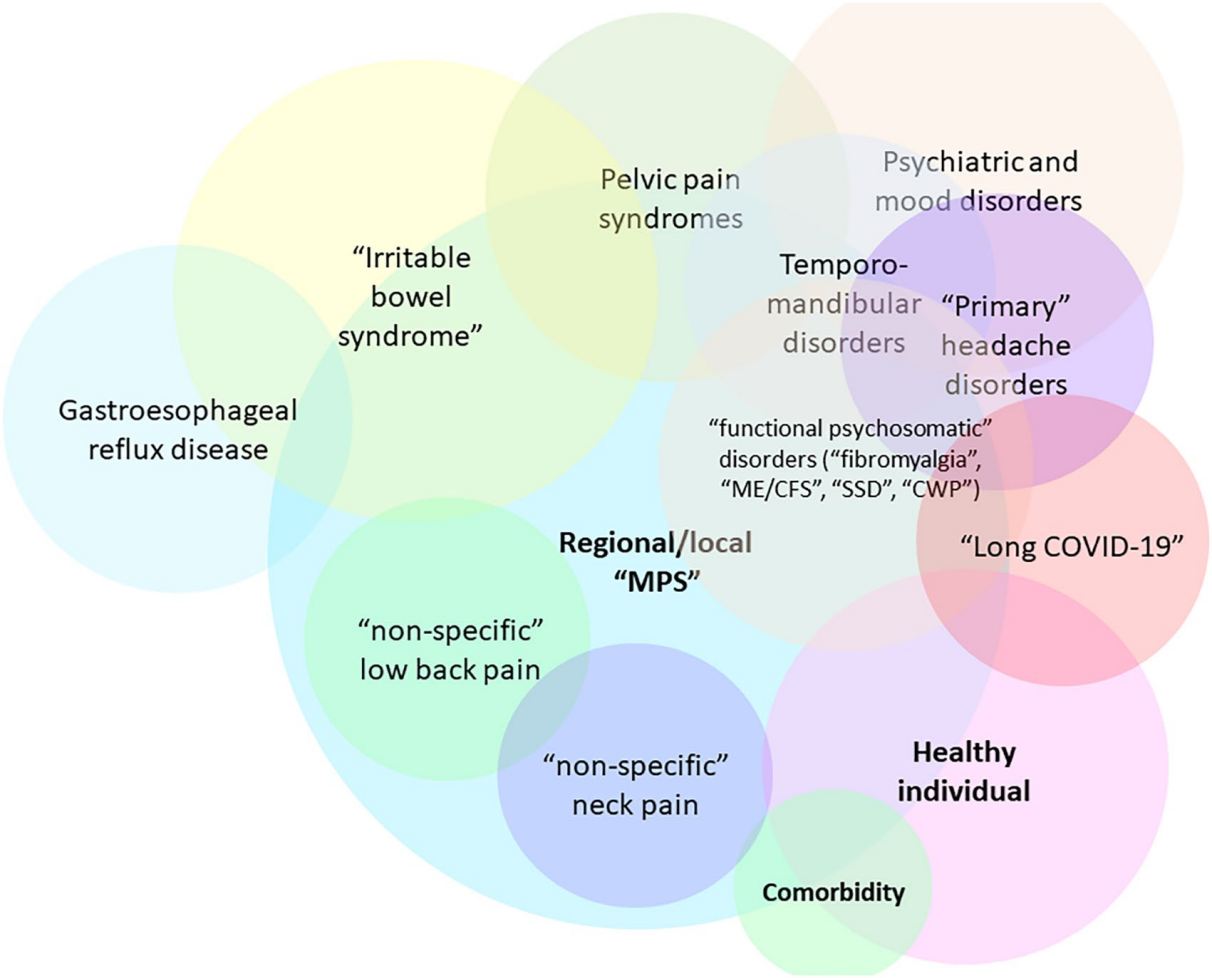
Overlaps of fibromyalgia-related and myofascial pain syndromes [adapted for long COVID-19 from Figure 7 in Plaut (2022) (4)]. Not all relationships and overlaps are depicted in this scheme. The colors have no special meaning. Variation in manifestation depends, in part, on the anatomical area and severity of involvement of the bio-tensegrity structure, and the downstream consequences of myofibroblast-generated-tensegrity-tension and fascial stiffness. If local fascial armoring becomes widespread, it will manifest as a fibromyalgia-like condition, depending on anatomical location, severity, and the layers involved, as well as additional soft tissue and visceral organ involvement. Psychological, metabolic, immune, endocrine, autonomic, behavioral, and other factors also contribute to variation in manifestation. The term “Healthy” is open to interpretation. “Nonspecific low back pain” reflects fascial armoring and imbalanced tensegrity forces in the lower back. “Nonspecific neck pain” reflects imbalance affecting the neck. “Primary headache disorders” i.e., imbalance in the head. “Irritable bowel syndrome” reflects fascial armoring and compressive forces in the lower back and abdomen (which might affect peristalsis and gut microbiome bidirectionally). “Myofascial pelvic pain syndrome” (including “interstitial cystitis”)-fascial armoring and fascial stiffness/pressure in the pelvic fascia, etc. If fascial armoring becomes more widespread, it can be expressed as somatic symptom disorder or fibromyalgia-like conditions. Long COVID-19 is included here in cases when this post infectious syndrome manifests as a fibromyalgia-like syndrome, with no clear pathology found, normal investigations and imaging and laboratory tests, and with marked discrepancy between patient complaints and clinical findings. CWP, chronic widespread pain; ME/CFS, myalgic encephalomyelitis/chronic fatigue syndrome; MPS, myofascial pain syndrome; SSD, somatic symptom disorder. Multiplying entities when there really is only one is not beneficial for science.

The central hypothesis presented in this work is that SARS-CoV-2 infection or acute COVID-19 can result in a myofascial and connective-tissue fibrotic disease by providing input (*via* immune signals and inflammatory mediators) into the positive feedback cascade of fascial armoring. This is hypothesized to lead to alterations in fascia’s remodeling mechanisms and a widespread induction of proto/myofibroblasts in myofascial and/or soft tissue extracellular matrix (ECM), mediating the transformation of fascial tissue. The cellular pathway of the pathophysiology of fibromyalgia was recently suggested to involve mechanosensitive signaling of myofibroblasts in soft tissue (e.g., integrin, focal adhesion, actin, Rho-associated-kinase, and transforming growth factor beta pathways) (4). Changes in the ECM have important effects on cells and physiological processes (6). Chronic tension in myofascial tissue and fascial dysfunction are expected to be hallmarks of fibromyalgia-like diseases (4). Therefore, due to its similarity with other functional somatic syndromes, “long COVID-19” is speculated to involve myofibroblast-mediated-biotensegrity-tension and mechanical compression in the fasciomusculoskeletal system along with immune-related processes (e.g., viral persistence, immune dysregulation, subclinical neuroinflammation, etc.). In severe cases, it would manifest as a global chronic exertional compartment-like syndrome (4, 7) impeding the function of various organ systems and hallmarked by multiorgan insidious progression as well as malaise and exertional fatigue. Figure 1 depicts the general overlap of conditions according to this framework where variation in manifestation depends, in part, on the overlap of anatomical areas of involved fascia and the severity of fascial armoring (4).

## 3. The theoretical model of “fascial armoring”

The “fascial armoring” model describes how contractile cells in myofascial tissue can help explain the symptomatology of fibromyalgia-like syndromes. In this section, an explanation of the model of “fascial armoring” is provided, as well as a description of cellular pathways linking COVID-19 to a myofascial bio-tensegrity abnormality. Empirical findings will be presented for explaining the model of fascial armoring with the purpose of suggesting a role for fascial myo/fibroblasts in “long COVID-19.”

### 3.1. A brief explanation of “fascial armoring” and its cellular pathway

The following findings are the basis for the theoretical model that is proposed in this work:

- Fascia is a continuous three-dimensional network of connective tissue that permeates the body at various layers and depths, connects and envelops organs and muscles, while undergoing constant remodeling and exhibiting qualities of tensegrity (or bio-tensegrity) (4, 6, 8, 9). According to the principle of tensegrity, structures are stabilized under conditions of continuous tension with discontinuous compression and behave as one connected structure (6, 10). “Bio-tensegrity” applies the principles of tensegrity to our understanding of the dynamics of living organisms (11). Fascia transmits forces to a distance through mechanical links and myofascial chains (9). In humans, most skeletal muscles are directly linked by connective tissue (9). Myofascial tissue is even continuous with the meninges (12).
- Fibroblasts are a main cell type synthesizing and regulating the ECM. When subjected to mechanical tension (and other signaling cues), fibroblasts differentiate into proto-myofibroblasts, a phenotype that contains cytoplasmic actin stress fibers terminating in fibronexus adhesion complexes (13, 14). These adhesion complexes serve to bridge the internal cytoskeleton of the proto/myofibroblast and its integrins with the surrounding ECM. The structural linkage created allows proto/myofibroblasts to generate traction and transfer contractile forces to the nearby ECM. Forces in the ECM are maintained and reinforced over time by matrix remodeling and further deposition of collagen and ECM material (13). The myofibroblast phenotype has increased expression of alpha-smooth muscle actin (α-SMA) fibers that enable them to maintain mechanical tension in the surrounding ECM in a mechanism known as slip and ratchet (13). A positive feedback regulation occurs with α-SMA and force generation, facilitated by transforming growth factor beta 1 (TGF-β1), and it is a basis for a vicious cycle that propels myofibroblast activity in granulation tissue, so cells can generate considerable amount of force. Myofibroblasts have similar cellular behavior independent of their anatomical location (15) although “fibroblasts” are a diverse family of cells (16, 17). It is possible that the protein conformation of α-SMA in myofibroblasts of fibrotic muscles differs from that of myofibroblasts of fibrotic lungs or in smooth muscle cells. Not all myofibroblasts express α-SMA (13) and using it as a marker for fibrogenic cell activity in skeletal muscle may be problematic (18).
- Myofibroblasts are found in the fascia and interstitial ECM of healthy individuals. They have a role in maintaining basal mechanical tissue tone (19, 20) and function as a large network (21). Soft tissue fibroblasts form a widespread interconnected cellular network with potentially critically overlooked physiological and functional importance (22).
- Fascia contains a dense network of sensory nerve endings that play a part in pain perception (23, 24).

The myofascial continuum in a tensegrity framework is the basis for the model of fascial armoring, which is portrayed as a global chronic compartment-like syndrome in severe cases. Empirical evidence that may support this fascia-based pathogenesis of fibromyalgia can be found in studies such as:

1. A study measured significantly higher intramuscular pressures in the trapezius muscle of fibromyalgia patients with a mean value of 33.48 ± 5.90 mmHg while the mean pressure measured in rheumatic disease controls was 12.23 ± 3.75 mmHg (25). The authors of that study note that the burden of the pressure abnormality in fibromyalgia might contribute to diffuse muscle pain and may be an intrinsic feature of the disease. The mean blood pressure is approximately 30 mmHg at the end of arterioles (26). Maintaining normal pressure gradients and starling forces is critical for proper perfusion in tissues and normal health. Chronic exertional compartment syndrome can be diagnosed when at least one of the following intramuscular compartment pressure measurements is measured: (i) pre-exercise pressure ≥ 15 mmHg (ii) 1-min post-exercise pressure of ≥30 mmHg, or (iii) 5-min post-exercise pressure ≥ 20 mmHg (27). Fibromyalgia as a disease of exclusively central neuroplastic pain should be reconsidered (25).
2. Fibromyalgia patients have decreased peripheral blood flow, which suggests there are functional disturbances in the cardiovascular system (28). There is evidence indicating a decreased transcapillary permeability in fibromyalgia patients (29) and abnormalities in arteries (30).
3. Biopsies of fibromyalgia cases indicate definite and nonspecific muscle changes which are suggested to result from chronic muscle contraction and ischemia of unknown etiology (31).
4. A study measuring muscle damping in fibromyalgia patients revealed elevated levels in all 14 sampled patients. This finding reflects increased muscle tension. The mean value in the fibromyalgia patient group was more than twice that of controls and maximum values were more than three-fold higher (32).
5. In muscles of fibromyalgia patients, increased deoxyribonucleic acid fragmentation and ultrastructural changes are suggested to be secondary to chronic muscle contraction (33).
6. A biopsy study of fibromyalgia patients found that peripheral fibroblast TGF-β gene expression is significantly higher compared to controls (34).
7. A study investigating inflammatory factors in skin biopsies from fibromyalgia patients showed elevated levels of interleukin 1β (IL-1β), in 38% of patients, IL-6 in 27%, and tumor necrosis factor-alpha (TNF-α) in 32% of patients. None of the cytokines could be detected in skin samples of healthy controls (35). These factors, along with TGF-β, are among the main mediators of myofibroblast activity (14, 36). Fibromyalgia patients have a unique cytokine profile of IL-6, IL-8, IL-10, TNF-α, and eotaxin/CCL24 (37).
8. The protein expression of genes involved in ECM turnover and oxidative metabolism has a differential expression in fibroblast of fibromyalgia patients, which could explain the inflammatory status of these patients (38).
9. Interstitial concentrations of metabolic substances in fibromyalgia were measured in a microdialysis study (39). Lactate, glutamate, and pyruvate concentrations were found to be significantly higher in fibromyalgia patients. Increased dialysate lactate in response to acetylcholine stimulation was shown in fibromyalgia (40).
10. Abnormalities in collagen crosslinking may contribute to remodeling of the ECM and deposition of collagen around the nerve fibers, which might lead to a lower pain threshold in fibromyalgia (41). A significantly lower amount of intramuscular collagen was found in fibromyalgia patients, which may cause a lower threshold for muscle micro-injury and result in non-specific signs of muscle pathology (42).

Even if myofibroblasts eventually de-differentiate to fibroblasts or undergo apoptosis, they would leave behind a remodeled dysfunctional fascia with altered biomechanical properties of the ECM. Tension and mechanical compression would lead to the activation of mechanoreceptors and nociceptors in nerve endings embedded in fascia, driving pain signals to the spinal cord and brain (4). Reducing the muscle pressure to normal levels in fibromyalgia may therapeutically alter the clinical picture (25). ECM forces causing stretch lesions in peripheral nervous tissue might affect sensory and sympathetic fibers, potentially causing further pain, radiation of pain, and autonomic abnormalities (4). The deep fascia is innervated with sympathetic nerve fiber endings (43), which suggests that the emotional and mechanical aspects of the disease are deeply integrated in the bio-tensegrity system.

Theoretically, chronic mechanical forces reaching the dura or intracranial/spinal fascia *via* myofascial chains and tensegrity dynamics might have significant physiological effects on the central nervous system. For example, mechanical stress increases amyloid beta, tau, and alpha-synuclein in the brain of wild type mice (44). Systemic circulating inflammatory mediators are expected to affect intracranial ECM and the brain. The downstream effects of chronic nociception due to fascial armoring would be similar to the effects of pain arising from other chronic painful conditions (e.g., increased neuroendocrine and hypothalamic–pituitary–adrenal axis activity, cortico-limbic and dopaminergic response to pain, brain neuroplasticity, sympathetic activity, microglia activation and possible excitotoxicity, central neuroinflammation, peripheral neurogenic inflammation, and so forth). While this paper did not focus on the central nervous system, it is noted that neuroendocrine and humoral pathways are part of fascial armoring and may help explain this complex entity. A more detailed mechanistic explanation of the above phenomena was provided in a recent review (4).

### 3.2. Elaboration on the molecular pathway linking inflammation to “fascial armoring” and induction of myofibroblasts

Upon activation, TGF-β1 promotes the formation and activity of myofibroblasts (45). Most of the TGF-β molecules are not synthesized on demand but are present in the ECM in latent form and can be activated when required (36). TGF-β1 can induce fibrosis by activating both canonical—small mother against decapentaplegic (SMAD) based and non-canonical (non-SMAD-based) signaling pathways. This, in turn, results in activation of myofibroblasts and superfluous production of ECM, while at the same time inhibiting ECM degradation (46). Non-SMAD pathways such as mitogen-activated protein kinase (MAPK), extracellular-signal-regulated kinase (ERK), JUN N-terminal kinase (JNK), RHO-associated kinase (ROCK), p38, and AKT pathways can also be activated by TGF-β1 (46, 47). SMAD2/3-SMAD4 is a heterodimer that often functions together with an AP-1 complex to mediate TGF-β-induced transcription. Upon cell damage and release of inflammatory substances from macrophages and T-cells, resident fibroblasts become activated by signals such as IL-6, TNF-α, and TGF-β. The activated fibroblast assumes a spindle shape and expresses high-affinity type 2 TGF-β receptor and begins to produce more collagen (47). Platelet derived growth factor (PDGF) has four isoforms that bind two PDGF receptor tyrosine kinases (PDGFR α and β). Fibroblasts and myofibroblasts express PDGFRs which can promote survival, proliferation, and migration when they bind their ligand PDGF (47). Mast cell degranulation of tryptase can induce myofibroblast differentiation and collagen synthesis directly and independently of TGF-β (48). Importantly, IL-1, IL-6, IL-11, IL-17, and IL-25, are cytokines that induce and enhance myofibroblasts (49). Along with integrins that can directly activate latent TGF-β induced pro-fibrotic pathways, inflammatory cytokines including IL-6 can have profound effects on the pro-fibrotic activity of fibroblasts (49).

Myofibroblasts fate is determined by complex mechanisms of cellular signaling. They can revert back to the fibroblast phenotype, or they can undergo apoptosis and self-clearance. Evasion from these mechanisms can lead to pathological and sustained fibrotic responses (47). Rho induces the nuclear translocation of Yes-associated protein/TAZ which promotes the expression of genes important for ECM, such as PAI-1, connective tissue growth factor (CTGF), and COL1. Stiffening of connective tissue directly drives fibrosis by controlling the integrin-mediated activation of latent TGF-β1. The mechanosensitive factor YAP-1 and its downstream signaling pathways of integrin beta-1 promote fibrosis (50). CTGF/CCN2 expression is induced by several stimuli including growth factors, cytokines, oxygen deprivation, mechanical stress, and more. CTGF/CCN2 has many functions (e.g., ECM turnover and cell behavior) and most TGF-β responses ultimately involve its stimulation (51).

Activation of certain toll-like receptors (TLRs) augments myo/fibroblast differentiation and fibrosis (52). Interleukin-1 receptor (IL-1R) kinase 4 (IRAK-4) has a phosphorylation activity that is essential for IL-1R/TLR-mediated cellular processes. A 2007 study showed the importance of IRAK-4 kinase activity in TLR-mediated myeloid differentiation factor 88-dependent signaling (53). That study investigated the single stranded ribonucleic acid (ssRNA) sensitive pattern recognition receptor TLR7. Also, neutrophil extracellular traps induce the epithelial to mesenchymal transition (54). Macrophages play a part in fibrotic changes in tissue and alter collagen crosslinking and matrix remodeling (*via* matrix metalloproteases, enzymatic secretion, paracellular signaling, macrophage-to-myofibroblast transition, etc.) (55, 56).

## 4. Long COVID-19

The following findings are presented with the purpose of outlining the manifestation of “long COVID-19” and the mechanism of COVID-19 induced tissue fibrosis.

### 4.1. Symptoms of “long COVID-19” and complications of SARS-CoV-2 infection

“Long COVID-19” is currently a recognized term in scientific literature. The Centers of Disease Control (CDC) and National Institute of Health (NIH) describe “long COVID-19” patients as individuals with lasting symptoms of COVID-19 that persist for more than 4 weeks since initial infection (1, 57). Manifestations of “long COVID-19” include:

#### 4.1.1. Fatigue and exertional fatigue

Unrelenting exhaustion, often reported as a constant state of weariness that reduces a person’s energy, motivation, and concentration, is common after COVID-19 (1) and may last more than 12 months after initial infection (58) often occurring independent of the severity of acute infection (58). “Long COVID-19” fatigue has much overlap with myalgic encephalomyelitis/chronic fatigue syndrome (ME/CFS) and many patients with “long COVID-19” fulfill the diagnostic criteria for ME/CFS (3). In a cross-sectional study, only 4% of COVID-19 patients had persistently abnormal findings on chest X-ray after recovery, while above 60 percent of patients did not feel back to full health more than 6 weeks after recovery from the acute phase (58).

#### 4.1.2. Dyspnea

Some patients with COVID-19 develop a fibrotic state in the lungs with ongoing dyspnea (1, 59). A study assessed the sequelae of COVID-19 at nearly 1 year following diagnosis while focusing on convalescent patients who had non-severe COVID-19 (60). Shortness of breath, fatigue, and sleep difficulties were among the more frequent symptoms. Pulmonary diffusion impairment was seen in 24% of non-severe cases. 56.7% of patients had abnormal CT findings, including ground-glass opacities, fibrosis, bronchiectasis, lines and bands, and nodules (60). Subjective respiratory symptoms are commonly reported after acute COVID-19 disease, but do not correlate with the severity of COVID-19 nor with pulmonary function (61).

#### 4.1.3. Cardiovascular abnormalities

A number of abnormalities can occur post COVID-19 (1): (i) Chronic inflammation of cardiomyocytes can cause myositis and death of cardiomyocytes; (ii) Autonomic abnormalities such as postural orthostatic tachycardia syndrome may arise due to dysfunction of the afferent autonomic nervous system; (iii) Fibroblasts in heart secrete ECM proteins when exposed to prolonged inflammation and cellular damage, resulting in fibrosis.

#### 4.1.4. Cognitive impairment and mental health

Cognitive impairment and psychiatric disorders are reported post COVID-19 (1). More than one third of patients who had non-severe COVID-19 reported anxiety and depression at approximately 1-year follow-up (60).

#### 4.1.5. More symptoms of “long COVID-19 syndrome”

More symptoms of “long COVID-19 syndrome” are cough, chest pain or chest tightness, myalgias, headache, sicca syndrome, rhinitis, poor appetite, diarrhea, dizziness (from orthostasis, postural tachycardia, or vertigo), alopecia, sweating, insomnia, sleep apnea, night sweats, restless legs, vivid dreams, nightmares, and lucid dreams (62–65). Olfactory and gustatory dysfunction are common in the chronic phase (1). Among the most frequent symptoms in COVID-19 survivors at the nearly 1-year follow-up, as found in a Chinese study investigating discharged patients hospitalized during the pandemic, are sleep difficulties (43.3%), shortness of breath (40.8%), fatigue (35.8%), and joint pain (32.5%) (60). Decreased quality of life in 44%–69% of adults was reported when measured 14–21 days after initial COVID test (64). In another study (65) conducted by an online survey across 56 countries, approximately 85% of participants experienced relapses primarily triggered by exercise, physical or mental activity, and stress. Participants experienced an average of 55.9 symptoms, across an average of 9.1 organ systems.

### 4.2. SARS-CoV-2 infection of various tissues and its related fibrosis

SARS-CoV-2 infection, similar to other infections, can lead to pro-inflammatory and pro-fibrotic processes with scarring and fibrosis of tissues (66). Various organs and tissues are susceptible due to the diverse viral tropism of SARS-CoV-2 (3, 67, 68). Myo/fibroblast induced fibrotic changes are seen in lungs, heart, liver, kidney, intestines, and several other organs following COVID-19 (48, 60, 67–75). Histopathological abnormalities after coronavirus infection are seen in integumentary, urinary, gastrointestinal, reproductive system, and more (69). Esposito et al. suggest that SARS-CoV-2 can directly invade the enteric and/or parasympathetic nervous system and spread along the vagus nerve or its afferents and innervations of visceral organs (76). Angiotensin converting enzyme 2 (ACE2) and TMPRSS2, the key mediators of SARS-CoV-2 infection (77), were identified in extrapulmonary and musculoskeletal tissue such as muscle cells, endothelial cells, smooth muscle cells, pericytes, muscle stem cells (satellite cells), macrophages, adaptive immune cells (B, T, or natural killer cells), myonuclei (muscle fibers), synovium cells, chondrocytes, and bone cells (osteoblasts and osteoclasts) (78–84). These findings suggest that the musculoskeletal system is a potential site of direct SARS-CoV-2 infection. Along with the multiorgan fibrotic impacts of COVID-19, myofascial tissue is also affected, as shown in the following findings:

#### 4.2.1. Adipocytes

ACE2 is expressed in adipose tissue (85) and there is evidence for replication of SARS-CoV-2 in adipocytes (74, 86). Adipose tissue is involved in SARS-CoV-2 infection in patients with older age, obesity, and diabetes (87). Adipocyte to myofibroblast trans-differentiation is induced by SARS-CoV-2 (88).

#### 4.2.2. Fibroblasts and ECM

SARS-CoV-2 mediates a dysregulation of the renin angiotensin aldosterone system and increased angiotensin II levels which might lead to several chronic and acute diseases. In COVID-19, fibroblast activation due to angiotensin II signaling is systemic, with possible involvement of lungs, heart, and kidneys (85). Another possible consequence of COVID-19 is a deregulation of the ECM homeostatic process, which can lead to excessive deposition and accumulation of disorganized ECM within end organs. ECM fibrotic changes alter the structure of the local tissue, disrupt the organ’s normal function, and can lead to dysfunction of an organ-system and eventually even organismal death (36, 89). SARS-CoV-2 infection increases mRNA levels of ACE2, TGF-β1, CTGF, and fibronectin-1, which are drivers of lung fibrosis (90).

#### 4.2.3. Skeletal muscle and myofascial tissue

Abnormalities in myofascial tissue may be part of COVID-19 and “long COVID-19.” Musculoskeletal injury and SARS-CoV-2 related viral myositis possibly results from direct viral invasion of myocytes or due to induction of autoimmune pathways (91). Viral invasion or a direct cytotoxic effect of coronavirus viral particles on skeletal muscle cannot be excluded (91). Viral particles were found in diaphragm and skeletal muscle postmortem following severe COVID-19 (92, 93). Systemic inflammation, hypoxemia, muscle disuse and immobilization, and malnutrition can also contribute to musculoskeletal impairment and fatigue following SARS-CoV-2 infection (91, 94). An autopsy series study of individuals with COVID-19 found signs of myositis (95). These inflammatory features were seen more in skeletal muscles than cardiac muscles. Patients with chronic COVID-19 had more pronounced inflammation although, overall, in most skeletal and cardiac muscles viral load was low or undetectable, suggesting inflammation arises due to circulating viral RNA rather than actual myocyte invasion. Therefore, it is suggested SARS-CoV-2 may be associated with a post-infectious immune-mediated myopathy (95). Another autopsy study of patients who died from COVID-19 found muscle and nerve tissue exhibited inflammatory/immune-mediated damage likely related to release of cytokines. Muscle atrophy, necrotizing myopathy, and myositis were found in psoas muscle samples. The same study found no evidence of direct SARS-CoV-2 invasion of these tissues (96). Histological sections show long COVID-19 patients have muscle fiber atrophy, metabolic alterations, and immune cell infiltration (91). Coronavirus causes strain and tension on skeletal muscles thus leads to myofascial trigger points (97).

Early evidence links “long COVID-19” to myofascial tissue and myofascial pain syndrome as a case report documented a patient with chronic symptoms of exertional dyspnea, brain fog, and myalgia post SARS-CoV-2 infection, with no previous medical history of chronic illness (98). He underwent an extensive multisystem workup that revealed normal pulmonary, cardiac, and end organ functions. His disabling fatigue and chronic pain at 6 months post COVID-19 were defined as “long COVID syndrome” relatively quickly, even though he also met the diagnostic criteria for fibromyalgia. Fibromyalgia Impact Questionnaire score was 88 out of 100 (i.e., severe fibromyalgia). Screening for depression was negative. Treatment with wet and dry needling was initiated for his myalgia and had good short-term therapeutic effects. Interestingly, after having multiple repeated sessions of needling in the torso and neck region achieving temporary pain relief and functional improvement, symptoms migrated/extended to the extremities and lower limbs at 6 months since having begun needling trial. Then, at 12 months since having COVID-19, after faithfully dry needling him in 10 different points with a 21-gauge 1 inch needle again repeatedly, his pain went into remission. This case report may indicate that “long COVID syndrome”-related myalgia might be a form of new-onset myofascial pain (98). Literature is still limited as studies of myofascial and subcutaneous tissue in “long COVID-19” are scarce, but it is unlikely that “central sensitization” mercifully withdrew in this case. A successful exorcism of fibromyalgia requires more than just needles. To the best of the author’s knowledge, no study was found to directly examine myofibroblasts in fascia following COVID-19.

## 5. Discussion

Plausible mechanisms of “long COVID-19” as suggested in literature are: (i) persistent reservoirs of SARS-CoV-2 in certain tissues, (ii) ongoing activity of primed immune cells, (iii) autoimmunity triggered by molecular mimicry, (iv) re-activation of neurotrophic pathogens such as herpesviruses under conditions of COVID-19 immune dysregulation, (v) SARS-CoV-2 interactions with host microbiome/virome communities and microbiome/virome dysbiosis, (vi) clotting/coagulation abnormalities, and (vii) dysfunctional brainstem/vagus nerve signaling (3, 99). These mechanisms may facilitate lasting “long COVID-19” symptoms and work in concert.

Findings from literature presented above show that SARS-CoV-2 and COVID-19 can lead to inflammation with systemically circulating inflammatory mediators and growth factors, and eventually to fibrosis in various tissues, including myofascial tissue. While induction of myofibroblasts normally occurs as a part of the inflammatory and healing process, their persistence in fascia, and the long-term consequences of ECM alterations during the acute and chronic phase, in terms of the fascial bio-tensegrity system, may have negative consequences on the organism. “Long COVID-19” and fibromyalgia-like syndromes such as ME/CFS share many characteristics. Some suggest they might share the same pathophysiology (100).

Along the discussion, the term “long COVID-19” is typically used by the author to refer to medically unexplained symptoms after COVID-19 (e.g., fatigue, irritability, cognitive and psychiatric symptoms, pain, etc.) that cannot be attributed to other medical conditions or comorbidities of the patient, and with no clear organic finding to explain them (no pulmonary fibrosis, no autoimmunity, no cardiomyopathy, no cachexia, no reactivated herpes virus, no thyroid disorder or other endocrine dysfunction, negative depression screening, normal clinical examinations, mostly normal routine laboratory tests and imaging investigations, etc.) and marked discrepancy between patient complaints and objective findings, usually labeled by the common clinician as “psychosomatic,” “catastrophizing,” or factitious.

### 5.1. Explaining symptomatology of “long COVID-19” in light of the model of “fascial armoring”

“Fascial armoring” as an entity resembling a mild global-chronic-compartment-like syndrome might help explain some known “long COVID-19” manifestations. Non-cardiac chest pain and chest tightness are common features of fibromyalgia and “long COVID-19.” Besides lung fibrosis, a fibrotic contractile fascial tissue in the trunk might be able to mildly impair lung function with possible chest tightness and wheezing. If a chronic mechanical compression develops in the cervical fascia or the superior cervical ganglion, it might disrupt salivary ducts/glands, perhaps leading to sicca. Contractile activity of myofibroblasts and subsequent mechanical tension in the temporal fascia could plausibly lead to a tension-like headache. In the abdomen—aberrant peristalsis and functional gut abnormalities. Pelvic fascia—genitourinary symptoms, and so forth. The main motif guiding the clinical interpretation of this entity throughout this discussion is mechanical tension, compression, and fascial stiffness.

#### 5.1.1. Cardiovascular abnormalities

Peripheral vascular alterations are seen in “long COVID-19” (101), including decreased flow mediated dilation and increased arterial stiffness (102, 103). Vascular abnormalities post COVID-19 were suggested to be due to an inflammatory vasculopathy (104). Vascular structural modification following SARS-CoV-2 infection may be due to the process of endothelial to mesenchymal transition in “long COVID-19” (105), which might increase the stiffness and pre-stress of the blood vessel wall. A global chronic exertional compartment-like syndrome would manifest with chronic fatigue, particularly so upon exertion. New onset of hypertension, as seen in “long COVID-19” might stem, in part, from widespread low-grade mechanical compression of blood vessels and increased resistance in skeletal muscle capillaries. Endothelial damage would result in release of tissue factor.

#### 5.1.2. Fasciomusculoskeletal, joint pain, and myalgia

Musculoskeletal pain in “long COVID-19” may be explained by increased intramuscular pressure, increased myofascial tension, and by sub/clinical chronic ischemia and elevated interstitial nociceptive substances (4). Alterations in skeletal muscle of “long COVID-19” are similar to ME/CFS (91). Potential pathological mechanisms leading to myofascial pain in SARS-CoV-2 infection include hypoxic muscle dysfunctions and psychological stress (97). A study suggested chronic proprioceptor activation might help explain pain in an experimental model of chronic fatigue syndrome (106). Morning stiffness-myofibroblasts contract over several hours to secure pre-stress and tension in ECM (13), therefore, after a period of rest and immobility a sense of stiffness should be more pronounced. Clinical features of fibromyalgia are common among those who had COVID-19 (107). In a study of 100 participants, fibromyalgia-like symptoms were studied in adult patients with “long COVID-19” *via* online questionnaires (108). 82% of patients were women; nine participants reported previous pain or an inflammatory condition. The most reported painful sites were chest, lower extremities, head/face, and migrating sites. Generalized pain was self-reported by 75% of participants. New onset fibromyalgia was suspected in 39 out of 100 “long COVID-19” patients, as they fulfilled the 2016 diagnostic criteria for “fibromyalgia.” Among the participants fulfilling the 2016 criteria, 23 were completely healthy prior to COVID-19. Many of “long COVID-19” patients develop pain, even if initial infection was mild, and there is an urgent need for pain management in “long COVID-19 syndrome” (108). Fascinatingly, “fibromyalgia” is often perceived as factitious/imagined by many clinicians who do not even believe this entity exists (4, 5). Even though the study above may suffer from biases such as recruitment bias and recall bias, perhaps leading to an overestimation of pain burden, it still deserves sincere consideration without dismissing the findings.

#### 5.1.3. Gastroesophageal reflux disease/acid reflux

Higher incidence of Gastroesophageal reflux disease (GERD)/acid reflux is seen following COVID-19 and has been noted as a manifestation of “long COVID-19” (109). Chronic mechanical disruption of gastroesophageal pressures and sphincter relaxations due to connective tissue compression, tension, and stiffening, might lead to GERD or a GERD-like condition.

#### 5.1.4. Coagulation abnormalities

Widespread fascial tensegrity compression would affect blood flow and turbulence in the vascular system as well as proper vasodilatation of vessels. Virchow’s triad consists of hypercoagulability, hemodynamic (turbulence/stasis), and endothelial injury/dysfunction. Therefore, coagulation abnormalities can be expected.

#### 5.1.5. Low-grade fever

Viral particle persistence and immune dysregulation are reasonable explanations for low-grade fever in “long COVID-19.” Chronic myofascial-related pain is associated with increased inflammatory mediators, serum reactive oxygen species, fibroblast growth factors, and neuroendocrine signaling (110, 111). Proinflammatory cytokines involved in COVID-19 and “long COVID-19,” such as IL-1 and IL-6, may contribute to the pathogenesis of fibromyalgia (107) and low-grade fever.

#### 5.1.6. Mood disorders

Post-traumatic stress disorder, anxiety, and depression are a manifestation of “long COVID-19” (109). Systemic fibroblast growth factors and other cytokines have an important effect on the brain (112). The intracranial ECM is suggested to have a role in neurological and psychiatric disorders (113–115). Stress (more precisely-restraint stress), arising from widespread myofibroblast-generated-tensegrity-tension in combination with psychological stress, is expected to lead to a wide range of pathologies. Interestingly, experimental models subjected to restraint stress reveal numerous abnormalities, e.g., depression and anxiety-like behavior, mechanical and cold allodynia, gut dysmotility, central nervous system abnormalities, infertility, metabolic abnormalities, and other phenomena (4, 116–125).

#### 5.1.7. Hair loss

By the effect of the myo/fibroblast network on the connective tissue sheath and follicular papilla and on hair cycling.

**Figure 2:**
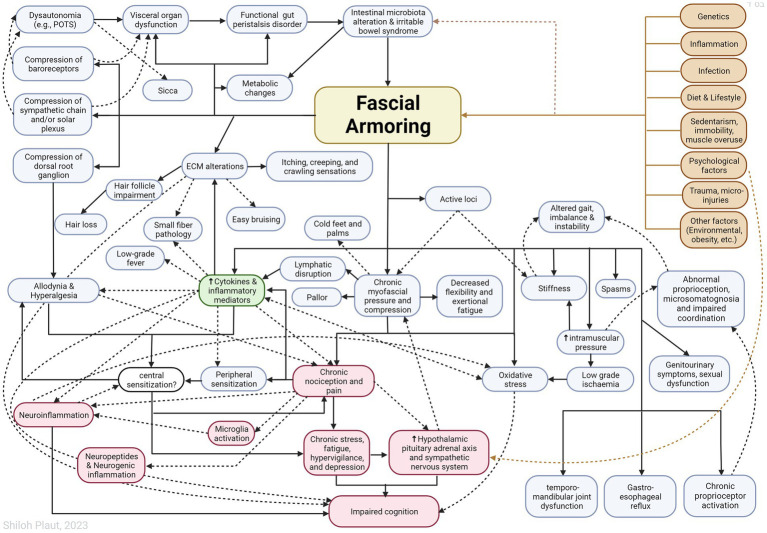
Outlining Fascial Armoring as a neuro-biomechanical medical entity in the form of bio-tensegrity tension and mechanical myofascioskeletal imbalance. Fascial armoring: myofibroblast-mediated-bio-tensegrity-tension and ECM abnormalities. Not all relationships are depicted with arrows in this scheme. Dashed arrows are meant for more visual clarity and have no special meaning. The occurrence of symptoms is not strictly dependent on the existence of pain. The clinical manifestation would depend on the anatomical location, structures involved, visceral organ involvement, and more. The theoretical model intrinsically has variations. ECM, extracellular matrix; POTS, postural orthostatic tachycardia syndrome. Figure created with BioRender.com, adapted from “flow chart” (2023), Available at: https://app.biorender.com/biorender-templates.

**Figure 3:**
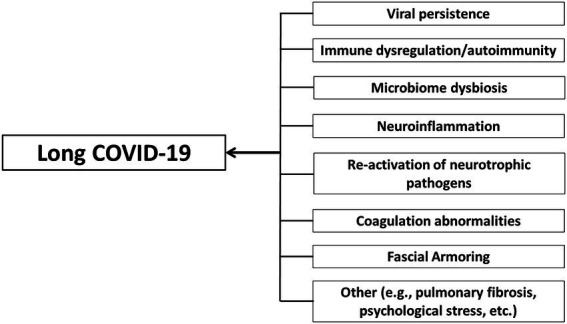
Long COVID-19 as a disease with a complex multifaceted pathophysiology that can involve multiple organ systems and tissues and an array of immune and non-immune processes. Multiple cellular mechanisms may contribute to long COVID-19. Fascial armoring is suggested to contribute to the symptomatology of long COVID-19 in cases when it manifests as a fibromyalgia-like syndrome. Although pulmonary fibrosis is mentioned, the term “long COVID-19” is generally used in the discussion to describe chronic medically unexplained symptoms after COVID-19 when no clear organic cause can be found to explain them, and with no comorbidity that can sufficiently account for them.

“Fascial armoring” will have different manifestations depending on the anatomical location and severity of the disease and visceral organ involvement. Some symptoms will evolve over time while others will resolve on their own. ECM remodeling and stress shielding is an ongoing dynamic process. Figure 2 outlines Fascial Armoring as a neuro-biomechanical medical entity characterized by bio-tensegrity tension, imbalance, and compression with multiorgan manifestations. While this work puts forward a theoretical model for part of the mechanism of “long COVID-19,” it is important to emphasize that this mechanism might be the explanation for only a subset of “long COVID-19” patients. This hypothesis and theory paper does not claim that the evidence for the suggested model is definitive or that all patients with persistent symptoms following COVID-19 have a fascial dysfunction. A connective tissue mechanism might be part of a broader mechanism or one of several parallel mechanisms occurring following COVID-19, as shown in Figure 3. Fibrotic changes and stiffening of lung tissue hinder gas exchange and result in compromised lung function leading to dyspnea and fatigue on exertion (49). Epigenetic changes are seen in “long COVID-19” and may contribute to age acceleration (126). Viral reservoirs or lingering fragments of viral particles in different tissues contribute to “long COVID-19” syndrome (72, 127).

### 5.2. Predicting findings in future “long COVID-19” investigations

Based on the model of myofibroblast-generated-tensegrity-tension and ECM alterations, predictions of results in future investigations of “long COVID-19” can be made as follows:

- Muscle damping measurements are expected to be higher in myalgic “long COVID-19” syndrome patients due to increased myofascial stiffness.
- In severe cases presenting as a fibromyalgia-like syndrome, increased intramuscular pressure is expected, depending on the site of the tensegrity pathology and imbalance. “Fascial armoring,” intrinsically, has anatomical and physiological variations (4).
- Elastography studies may be used to quantify pre and post infection values of shear wave measurements and stiffness/rigidity of fascia in different anatomical sites. Elasticity and rigidity of the fascia can be measured, and the stress–strain relationship and stress relaxation under uniaxial elongation. Load-strain and load-time curves, hysteresis, and stress-relaxation tests should find differences between severe and mild cases of “long COVID-19,” and between patients and healthy controls.
- Microdialysis studies can be used to quantify signs of sub/clinical hypoxia in tissues.
- Dermatological manifestations are expected: abnormal ECM properties could predispose to easy bruising. ECM internal forces and contractility of myofibroblast populations under the skin, would lead to creeping, crawling, and itching sensations by activating various sensory receptors embedded in superficial fascia and skin. Small fiber neuropathy may be expected as well. Besides the effects of the acute inflammatory phase of COVID-19, compression and stiffening of dermal and subcutaneous (superficial) fascial tissue could affect small neuronal fibers, perineurium, and Schwann cells. The effect of tissue stiffness and mechanical load on neuronal axons (128, 129) in this model was explained elsewhere (4)
- Ultrasound doppler can be used to measure altered blood flow in vessels. Pulse wave velocity can be used to estimate arterial stiffness. Results are expected to reflect a mild–moderate fascial compressive disorder.
- Pallor would be an overlooked manifestation in fibromyalgia-like cases. Chronic compressive forces in the periorbital fascia would lead to reduced optic disc perfusion.
- Biopsies can be taken to sample fascia from various sites, however, these affect the tensegrity structure and, if done, should be done cautiously to minimize unnecessary iatrogenic harm. Fibromyalgia patients were found to have several fascial and ECM abnormalities, including increased TGF-β gene expression in biopsy samples of peripheral fibroblasts, as well as elevated IL-1 and IL-6 levels (34, 35, 38, 41, 130, 131). Similarly, widespread disorganization of subcutaneous ECM fibers would be a “non-specific” finding on biopsies of fascia in “long COVID-19.” Concentration of myofascial myofibroblasts and α-SMA should be higher. Proto-myofibroblasts are an intermediate form of cells in the fibroblast-to-myofibroblast transition, and they do not contain as much α-SMA yet do generate mechanical force. Muscle biopsy from chronic cases will reveal moth-eaten appearance and ragged red fibers and changes due to low-grade chronic ischemia.

To the best of the author’s knowledge, current research has yet to fully examine or study most of the above investigations in “long COVID-19.”

### 5.3. Predicting risk factors and protective/relieving factors

Based on the presented model, several factors are predicted to worsen/predispose or relieve “long COVID-19” and fibromyalgia-like syndromes. For example:

- Patients with hypermobility syndrome are expected to be at significantly increased risk of “long COVID-19” and “fibromyalgia.” This association is predicted because collagen microarchitecture affects mechanosensitive signaling and can induce fibroblast-to-myofibroblast differentiation. Studies have shown that patients with hypermobility syndrome have widespread ECM disarray and increased myofibroblasts (4, 132). In our context, an imbalanced tensegrity structure with laxity in its frame is expected to collapse more easily.
- Sedentary and physically inactive persons (for example, during lockdowns or isolation): sedentarism is suggested to be a major factor affecting myofascial pain/fibromyalgia syndrome because immobility and disuse lead to muscle fibrosis and atrophy (4, 133).
- Inflammatory conditions contribute pro-inflammatory and pro-fibrotic input into the fascial armoring mechanism, therefore, “long COVID-19” is expected to be associated with other chronic inflammatory and autoimmune disorders.
- Individuals that tend to wear tight clothes and accessories are predicted to have a higher odds ratio/relative risk for “long COVID-19” because of the chronic mechanical input these factors provide to the myofibroblast cascade of contracture. A study suggests mechanical tension on skin induces myofibroblasts (134). According to another study, applying mechanical stimuli in the form of a splint induces myofibroblasts (135). Tight-fitting clothes are suggested to promote myofascial pain (4, 136, 137).
- Low vitamin D might predispose to disease. Vitamin D is involved in myofibroblast de-differentiation (138).

Obesity, a history of “psychosomatic” or “nonspecific” illness, and a low estrogen state (polycystic ovary syndrome, turner syndrome, post-menopausal women, men, etc.) might be predisposing factors for causing more severe or persistent disease:

- Obesity and diabetes as a risk factor may be deduced from a study (139) showing that connective tissue fibrosis induced by obesity is dependent on mechanosensitive signaling. Myofibroblast-mediated fibrosis can change the plasticity of subcutaneous tissue and increase connective tissue rigidity and stiffness (139). The effect that chronic injections have on the tensegrity system might augment the disorder.
- Under this framework, a previous medical history of chronic myofascial pain or a functional psychosomatic disorder in an individual indicates the presence of “facial armoring” in fascia and is, therefore, anticipated to be a risk factor.
- Contraceptive pills are expected to be a protective factor against “long COVID-19.” It is suggested that higher incidence of fibromyalgia is not necessarily associated with abnormal estrogen levels (140, 141). Fibroblasts express hormone receptors (142, 143). Estrogen has an inhibitory effect on myofibroblast differentiation and is also shown to be associated with lower fascial stiffness (144, 145). Hormonal contraceptives are associated with reduced risk of fibromyalgia in women (146). Estrogen receptor-β, Sirt1, and hydrogen sulfide can diminish TGF-β signaling (45). A study conducted by survey sampled 616 individuals (77.4% women) found new onset of fibromyalgia symptoms after COVID-19 infection occur more often in males (OR: 9.95, 95% CI 6.02 to 16.43, *p* < 0.0001) and in obesity (OR: 41.20, 95% CI 18.00 to 98.88, *p* < 0.0001) (107), although one should take into account that online surveys have intrinsic limitations and biases such as self-selection bias (107). The sex distribution of “long COVID-19” would depend on estrogen levels as well as multiple other factors including lifestyle factors and other (genetic, environmental, behavioral, etc.). Interestingly, with obesity being such a strong associated predictor of fibromyalgia after COVID-19 (OR > 40), the intensity of treatment setting had odds ratio of only ~1.7, pre-existing anxiety had an odds ratio of ~1.3 (95% CI 0.858 to 2.013), and pre-existing depression had odds ratio of 1.255, *p*-value = 0.507. Meanwhile, neurobiopsychosocial theories for fibromyalgia put a major emphasis on stress and psychology.

**Figure 4:**
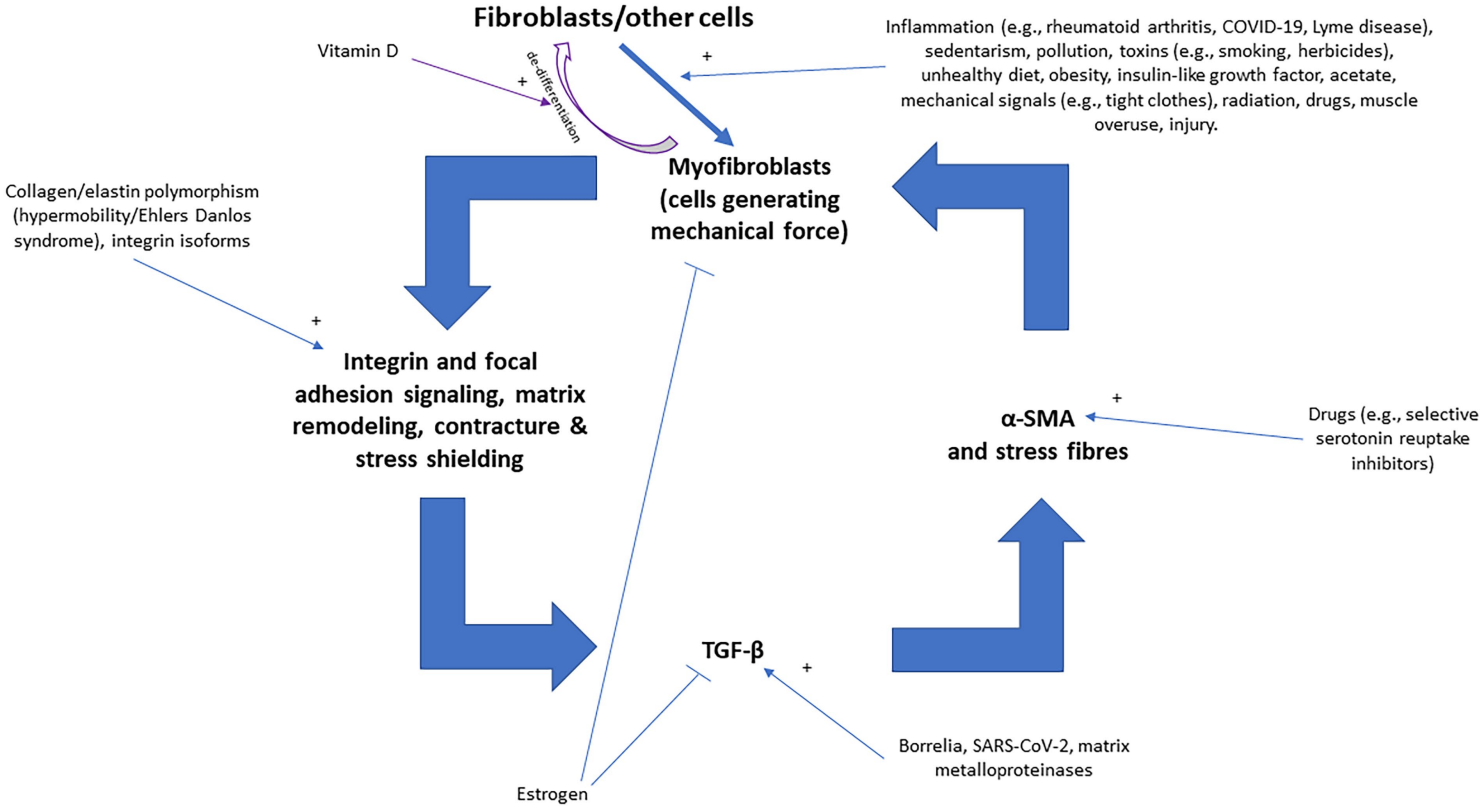
The positive feedback loop of fascial armoring (fascial rigidity and tensegrity tension) with inputs into the vicious cycle of myofibroblasts [adapted for long COVID-19 from Figure 3 in Plaut (2023) (7)]. Fibroblasts, adipocytes, and other cell-types can differentiate to myofibroblasts (the intermediate proto-myofibroblast phenotype is not shown here). Empirical studies show that various factors influence fibroblast-to-myofibroblast differentiation (and epithelial to mesenchymal transition), including immobility, infection/inflammation, diet, herbicides, drugs, epi/genetics, etc. (4, 138, 144). Once myofibroblasts activity is initiated, a positive feedback loop can stimulate and maintain fascial armoring, i.e., lead to a chronic fascial bio-tensegrity pathology driven by a myo/fibroblast network of contracting cells in connective tissue. Any imbalance in the constant interplay of the various promoting/inhibiting factors in this loop will regulate the course of the disease. Macrophages and epithelial-to-mesenchymal transition can also lead to (or differentiate to) myofibroblasts but are not depicted in this scheme for the purpose of simplicity. Open arrows with a plus sign indicate upregulation/stimulation, closed arrows indicate downregulation/inhibition. TGF-β, transforming growth factor beta; α-SMA, alpha smooth muscle actin.

Figure 4 illustrates various inputs into the positive feedback loop of fascial armoring, where factors that stimulate or inhibit myofibroblasts provide their input. The theory of “fascial armoring” links between unhealthy lifestyle (e.g., diet, sedentarism, smoking etc.) and pain and suffering. As with most conditions, it is multifactorial. Lifestyle interventions and exercise (movement), therefore, may have a major role in the treatment of “long COVID-19” and in psychosomatic syndromes. A study showed cyclical mechanical stress reduces myofibroblast differentiation of primary lung fibroblasts (147). Movement might help release fascial tensions by various mechanisms (4) and immobility leads to fibrosis and increase in myofibroblasts (4, 133). Needling therapy (when done correctly) is expected to modulate the disease course because of its suggested effects on the bio-tensegrity system (7).

## 6. Conclusion

“Long COVID-19” exhibits fibromyalgia-like manifestations and symptomatology including chronic fatigue, cognitive impairment, low mood, functional impairment, and last but not least-myofascial pain (i.e., “fibromyalgia-ness”). Globally, the long COVID-19 wave has risen and is leading to extensive morbidity, decreased quality of life, and suffering in society. SARS-CoV-2 infection can lead to pro-inflammatory and pro-fibrotic responses in multiple tissues, including myofascial tissue, and leads to fibrosis and upregulation of myofibroblasts. Multiple parallel mechanisms might be at work in long COVID-19 syndrome, which is hypothesized in this manuscript to involve a chronic connective tissue pathology and changes in the myofascioskeletal bio-tensegrity system as well as immune dysregulation, viral persistence, immune-mediated myopathy, muscle atrophy, and other abnormalities. Further research is required to enhance our understanding of the underlying mechanisms of- and the extent of soft tissue damage in- “long COVID-19 syndrome.” The coronavirus pandemic offers us an opportunity to better understand peripheral mechanisms of “functional psycho/somatic syndromes” such as “fibromyalgia,” “ME/CFS,” “post Lyme disease,” and related conditions. The boundary of a medical entity is determined by the boundary of the biological process that underlies it. This theoretical model offers one mechanical aspect as part of a common immuno-rheumo-psycho-neurological mechanism. Biology does not separate itself into different medical specialties, and the body and the mind are one flesh, one Being. The principle of Occam’s razor (i.e., “medical monotheism”) favors minimizing the multiplication of entities. According to fascial armoring, indeed, there is only one entity…

## Data availability statement

The original contributions presented in the study are included in the article/supplementary material, further inquiries can be directed to the corresponding author.

## Author contributions

SP solely contributed to the study conception and design, material preparation, data collection, data curation, conceptualization, integration of information, formal analysis investigation, methodology, visualization, resources, writing—original draft, and writing—review and editing.

## Conflict of interest

The author declares that the research was conducted in the absence of any commercial or financial relationships that could be construed as a potential conflict of interest.

## Publisher’s note

All claims expressed in this article are solely those of the authors and do not necessarily represent those of their affiliated organizations, or those of the publisher, the editors and the reviewers. Any product that may be evaluated in this article, or claim that may be made by its manufacturer, is not guaranteed or endorsed by the publisher.

## Figures and Tables

**Table 1:** 

ACE	Angiotensin converting enzyme
CDC	Centers of Disease Control
CI	Confidence interval
COVID-19	Coronavirus disease 2019
CWP	Chronic widespread pain
CTGF	Connective tissue growth factor
DNA	Deoxyribonucleic acid
ECM	Extracellular matrix
ERK	Extracellular-signal-regulated kinase
GERD	Gastroesophageal reflux disease
GPNF	Global percutaneous needle fasciotomy
IL	Interleukin
IL-1R	Interleukin-1 receptor
IRAK-4	Interleukin-1 receptor kinase 4
JNK	JUN N-terminal kinase
MAPK	Mitogen-activated protein kinase
ME/CFS	Myalgic encephalomyelitis/chronic fatigue syndrome
MLCK	Myosin light chain kinase
NICE	National Institute for Health and Care Excellence
OR	Odds ratio
PDGF	Platelet derived growth factor
ROCK	Rho-associated protein kinase
SARS-CoV	Severe acute respiratory syndrome coronavirus
SMA	Smooth muscle actin
Smad	Small mother against decapentaplegic
SSD	Somatic symptom disorder
ssRNA	Single stranded ribonucleic acid
TAZ	Transcriptional coactivator with PDZ-binding motif
TGF-β	transforming growth factor beta
TLR	Toll-like receptor
TNF-α	tumor necrosis factor alpha
YAP-1	Yes-associated protein 1
